# Optical properties of a hexagonal C/BN framework with sp^2^ and sp^3^ hybridized bonds

**DOI:** 10.1038/s41598-020-63693-2

**Published:** 2020-04-22

**Authors:** Hongxia Bu, Haibin Zheng, Hongyu Zhang, Huimin Yuan, Jingfen Zhao

**Affiliations:** 1 College of Physics and Electronic Engineering, Qilu Normal University, Jinan, Shandong 250200 China; 2 Department of Physics, East China University of Science and Technology, Shanghai, 200237 China

**Keywords:** Physical chemistry, Chemical physics

## Abstract

We investigated the optical properties and roles of sp^2^- and sp^3^-hybridized bonds of a hexagonal C/BN family using first-principles calculations. The calculated phonon dispersions confirm the dynamic stability of Hex-(BN)_6_C_12_ and Hex-C_12_(BN)_6_. The complex dielectric function evolves from the infrared to the ultraviolet region and has a significant anisotropy for different polarizations. The reflectivity and refractive index spectra show that the sp^2^-hybridized C atoms are more sensitive to the light from infrared to visible region than B-N pairs while the C atoms and B-N pairs have a similar sensitivity to high frequencies. The sharp peaks of the energy-loss spectrum are all concentrated in the 23–30 eV energy region, which can be used to identify these hexagonal structures. The calculated band structures show Hex-C_24_ and Hex-(BN)_6_C_12_ are metals, but Hex-C_12_(BN)_6_ and Hex-(BN)_12_ are semiconductors with indirect band gaps of 3.47 and 3.25 eV, respectively. The electronic states near the Fermi level primarily originate from sp^2^-hybridized atoms. In addition, sp^2^-hybridized bonds are the main elements affecting the optical and electronic structure of C/BN materials with sp^2^- and sp^3^-hybridizations. We expect that the results presented will help understand the optical properties of C/BN materials containing sp^2^- and sp^3^-hybridized C atoms and B-N pairs.

## Introduction

Boron (B), carbon (C), and nitride (N) atoms are in the same row of the periodic table of elements and all have the flexibility of sp, sp^2^, and sp^3^ hybridizations. B, C, and N atoms form abundant frameworks in simple substances or compounds. In addition, B and N have a similar atomic radius to C, and B-N pairs have the same number of outer shell electrons as C-C. Since the emergence of new carbon allotropes in 1980, different methods were proposed to experimentally synthesize or theoretically investigate a large number of BN analogues of carbon allotropes, such as diamond-like C-BN, fullerene-like BN nanocages^1–3^, BN nanotubes^4–7^, BN nanosheets^8–12^, BNyne^13^, M-BN^14^, Z-BN^15^, and O–BN^16^, etc. Among them, C-BN, BN nanocages^1,2^, BN nanotubes^4,5^, BN nanosheets^8–12^ have been synthesized experimentally by various methods and BNyne^13^, M-BN^14^, Z-BN^15^, and O–BN^16^ are still in the stage of theoretical prediction. Anyway, there are some similar properties between the BN materials and the known carbon materials. For instance, graphite and h-BN are widely used as lubricant, while diamond and c-BN are both super-hard materials. In contrast, some properties of the BN materials differ significantly from their carbon counterparts because of the fundamental difference between the polar covalent bond (B–N bond) and the pure covalent bond (C–C bond). For example, the electronic structure of C/BN bi-ribbons with zigzag-edged graphene and BN nanoribbons can transform from a semiconductor to a half-metal and to a ferromagnetic metal when the C ratio increases^17^. This produces interesting results that these C/BN materials often have structural similarities but radically different thermal conductivity, chemical stability, electronic, and optical properties. Considerable efforts have been devoted to the research on various C/BN structures motivated by their intriguing properties.

Chemical doping with B-N pairs is the most common method to adjust the physical and chemical properties of carbon nanomaterials. Xu *et al*. reported that the band gap increases with the increase of BN nanodots in the BN-doped graphene superlattice^18^. Zhou *et al*. discussed the modulation of the band gap in BN-yne structures composed of hexagonal BN rings joined by C-chains with a band gap from 2.65 eV for n = 2 to 1.14 eV for n = 12 where n is the number of C atoms in the chain^19^. Our work also shows that the structural stability and electronic properties of graphdiyne are relevant to the BN doping rate and position^20^. The influence of B-N pairs doping in carbon materials on the optical properties has become an important aspect of many studies. The electronic and optical properties of 6,6,12-graphyne can be altered by co-doping with B-N pairs^21^. By doping with BN at different sites, Bhattacharya *et al*. pointed out that the optical band gap of graphyne can be tuned from the infrared to the ultraviolet (UV) region^22^. They also studied the optical properties of γ-GNTs and their BN analogues (γ-BN-yne)^23^. They showed that the tubular geometry rather than its parent planar structure is the main factor to determine the optical properties of γ-GNTs. And the structure of γ-BN-yne is suitable for applications in novel optoelectronic devices. Yun *et al*. presented the optical properties of graphyne-like BN nanotubes^24^. However, the optical properties and the roles of sp^2^ and sp^3^ hybridized bonds have been rarely used for 3D multi-porous C/BN polymorphs compared with one-dimensional nanotubes (NTs) and two-dimensional nanosheets.

**Figure 1:**
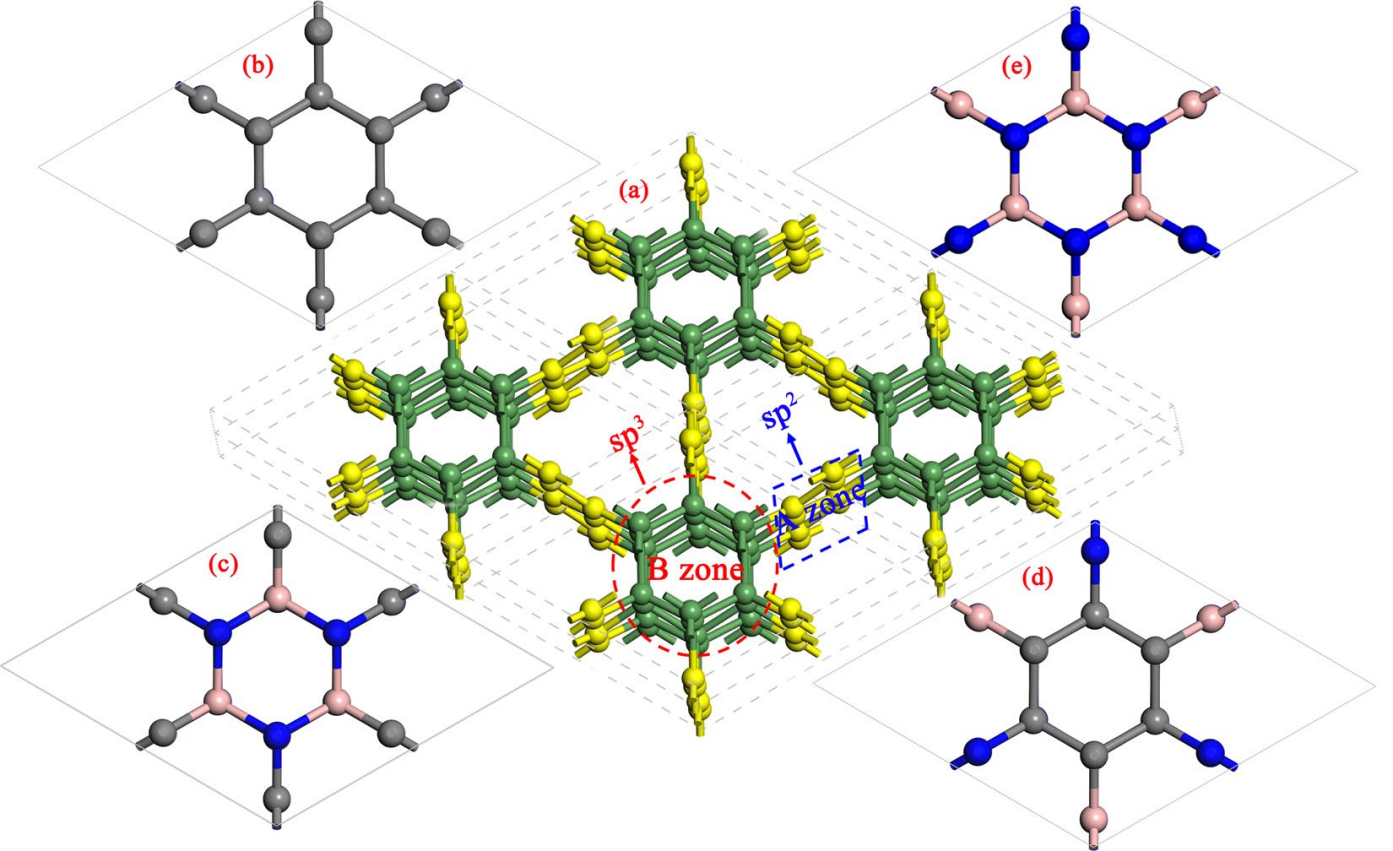
Atomic structure of the hexagonal C/BN family. (**a**) schematic model of the (2 × 2 × 2) super-lattice formed from sp^2^-hybridized (yellow balls, A zone) and sp -hybridized atoms (green balls, B zone). (**b–e**) are detailed atoms structures of (**b**) Hex-C_24_, (**c**) Hex-(BN)_6_C_12_, (**d**) Hex-C_12_(BN)_6_, and (**e**) Hex-(BN)_12_. The grey, pink, and blue atoms represent C, B, and N atoms, respectively.

Given the importance of optical properties, we systematically studied the optical properties of B and N doped Hex-C_24_^25^ using first principle calculations. The doped structures form a hexagonal C/BN family with sp^2^ and sp^3^ hybridized bonds. Previous works indicate that nearest neighbor position is the most stable structure of B and N atoms doped into graphene^26^. Therefore, we followed this rule and considered four configurations: pristine Hex-C_24_, B-N pairs doping sp^3^ hybridized carbon atoms (Hex-(BN)_6_C_12_), B-N pairs doping the sp^2^ hybridized carbon atoms (Hex-C_12_(BN)_6_), and B-N doping all carbon atoms to form pristine Hex-(BN)_12_, as shown in Fig. 1. To get insights into the optical properties of these C/BN networks, we investigated the dielectric function, the absorption coefficient (α(ω)), the reflectivity(R(ω)), the refractivity index ((n(ω)), and the energy-loss(L(ω)). We established helpful guidelines for the applications of such hexagonal C/BN frameworks.

## Results

The crystal structures of Hex-C_24_ and Hex-(BN)_12_ were determined earlier^25,27^. In order to minimize the symmetry reduction, we used B-N pairs to dope the sp^3^ hybridized carbon atoms (named as Hex-(BN)_6_C_12_), B-N pairs to dope the sp^2^ hybridized carbon atoms in pristine Hex-C_24_ (named as Hex-C_12_(BN)_6_), and B-N to dope all the carbon atoms forming pristine Hex-(BN)_12_. The four crystal structures are shown in Fig. 1. For Hex-(BN)_6_C_12_, the lattice parameters are a = b = 6.987 Å and c = 4.248 Å, and the corresponding values of Hex-C_12_(BN)_6_ are a = b = 6.950, Å and c = 4.240 Å. The values of lattice parameters are between that of Hex-C_24_ and Hex-(BN)_12_. There are very small distortions of about 2% compared with Hex-C_24_. For these two structures, they both have four different chemical bonds: C-C, C-N, C-B, and B-N. The corresponding bond lengths of Hex-(BN)_6_C_12_ are 1.406 and 1.371 Å (in the axis direction) for sp^2^-C-C-sp^2^, 1.486 Å for sp^2^-C-N-sp^3^, 1.577 Å for sp^2^-C-B-sp^3^, and 1.607 and 1.584 Å (in the axis direction) for sp^3^-B-N-sp^3^. And that values of Hex-C_12_(BN)_6_ are 1.565 and 1.563 Å (in the axis direction) for sp^3^-C-C-sp^3^, 1.481 Å for sp^3^-C-N-sp^2^, 1.580 Å for sp^3^-C-B-sp^2^, and 1.421 Å for sp^2^-B-N-sp^2^. Obviously, for B, C, and N elements, the bond length difference of the same hybrid form is smaller in this hexagonal C/BN family.

To evaluate the relative stability of this hexagonal C/BN family, we calculated the cohesive energy (*E*_*coh*_) using the definition:

$${E}_{coh}=\frac{m{E}_{C}+n({E}_{B}+{E}_{N})-{E}_{{C}_{m}{(BN)}_{n}}}{m+2n}$$

where *E*_*C*,_ *E*_*B*_, *E*_*N*_, and *E*_*Cm(BN)n*_ are the energies of a single C atom, B atom, N atom, and the total energy of the corresponding C/BN configuration, respectively. The calculated results show that the cohesive energy of Hex-C_24_, Hex-(BN)_6_C_12,_ Hex-C_12_(BN)_6,_ and Hex-(BN)_12_ are 7.817, 7.560, 7.608, and 7.733 eV per atom, respectively. From the cohesive energy values, we can see the order of energy stability is Hex-C_24_, Hex-(BN)_12,_ Hex-C_12_(BN)_6,_ and Hex-(BN)_6_C_12_. Hex-C_12_(BN)_6_ is energetically favored over Hex-(BN)_6_C_12_, which means B-N pair prefers to replace the sp^2^-hybridized carbon atoms under these conditions.

**Figure 2:**
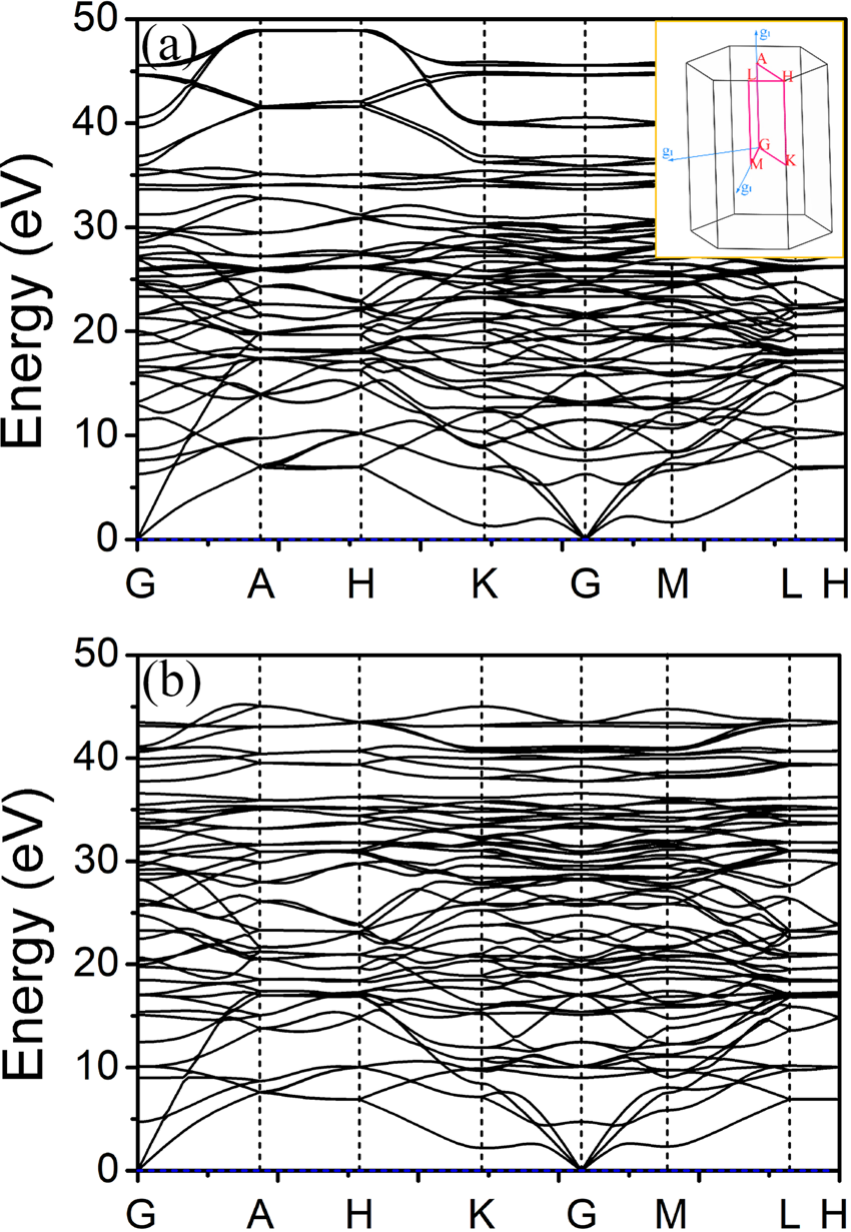
Phonon band structure in (**a**) Hex-(BN)_6_C_12_ and (**b**) Hex-C_12_(BN)_6_.

To the dynamic stability of Hex-(BN)_6_C_12_ and Hex-C_12_(BN)_6_, we calculated the phonon dispersion using finite displacement theory, as shown in Fig. 2. No imaginary frequencies are observed along the high symmetry orientations in the Brillouin zone, which confirms the dynamic stability of Hex-C_24_ and Hex-(BN)_12_.

**Figure 3:**
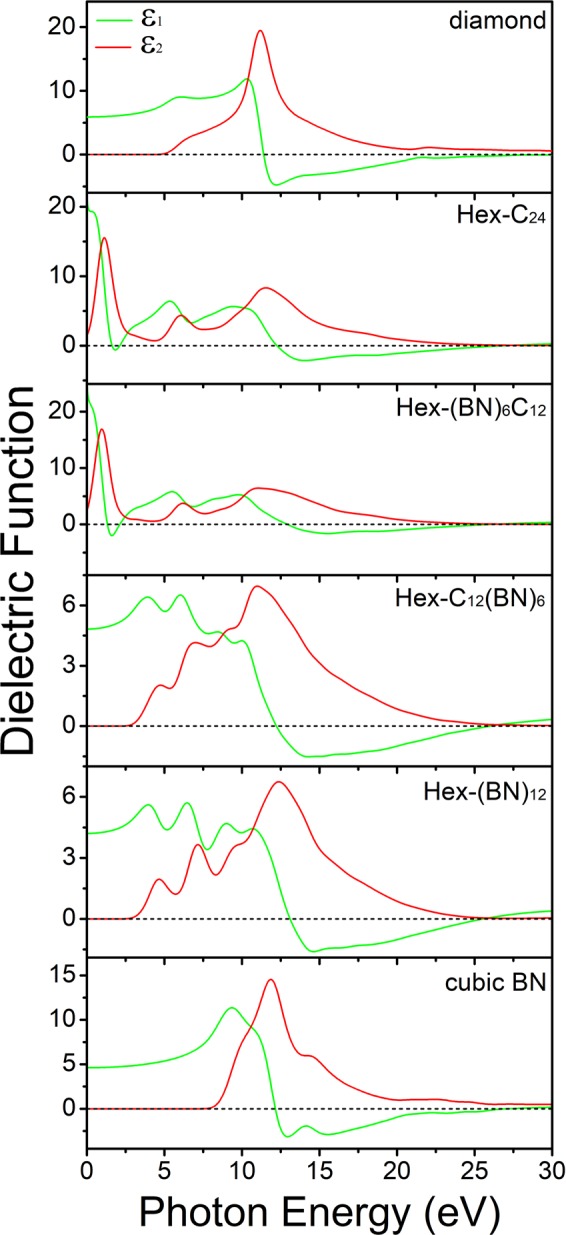
Dielectric function of Hex-C_24_, Hex-(BN)_6_C_12_, Hex-C_12_(BN)_6_, and Hex-(BN)_12_ polycrystalline structures. The values of diamond and cubic BN are shown for comparison.

We calculated the dielectric functions of polycrystalline Hex-C_24_, Hex-(BN)_6_C_12,_ Hex-C_12_(BN)_6,_ and Hex-(BN)_12_. The complex dielectric function of diamond was calculated first and was in good agreement with the experimental data^28^. Then, the complex dielectric function of the hexagonal C/BN framework for poly-crystals were obtained similarly, as illustrated in Fig. 3. For comparison, the complex dielectric functions of the diamond and cubic BN were also calculated. We observed that the complex dielectric functions are tuned from the infrared to the UV region of the electromagnetic spectra for the hexagonal C/BN family. Compared to diamond and cubic BN which have a cubic symmetry with sp^3^ hybridized atoms, the first peaks of ε_1(ω)_ and ε_2(ω)_ of these four hexagonal C/BN frameworks have significant redshifts. Hex-C_24_ has a spectrum similar to that of Hex-(BN)_6_C_12_, whereas Hex-C_12_(BN)_6_ has a spectrum similar to that of Hex-(BN)_12_. In Hex-C_24_ and Hex-(BN)_6_C_12_, in which all the atoms of sp^2^ hybridization are carbon atoms, the spectra of the complex dielectric function are narrow and sharp in the low-energy region. In Hex-C_12_(BN)_6_ and Hex-(BN)_12_, in which all the atoms of sp^2^ hybridization are B-N pairs, the complex dielectric functions are broad and the intensity decreases. Compared with Hex-C_24_ and Hex-(BN)_6_C_12_, the ε_1(ω)_ peak and the ε_2(ω)_ edges of Hex-C_12_(BN)_6_ and Hex-(BN)_12_ have significant blueshifts, which means that they have larger band gaps. The significant blueshifts of the ε_2_(ω) spectra due to the presence of C/BN with sp^2^ hybridization at different sites matches well with other C/BN molecules^23,29^. The sp^2^ hybridization has a significant impact on the dielectric function of the materials with sp^2^ and sp^3^ hybridized C atoms and B-N pairs. Because of the shift to the UV region and the low dielectric constant of Hex-C_12_(BN)_6_ and Hex-(BN)_12_, they are suitable candidates for short-wavelength optoelectronic devices.

**Figure 4:**
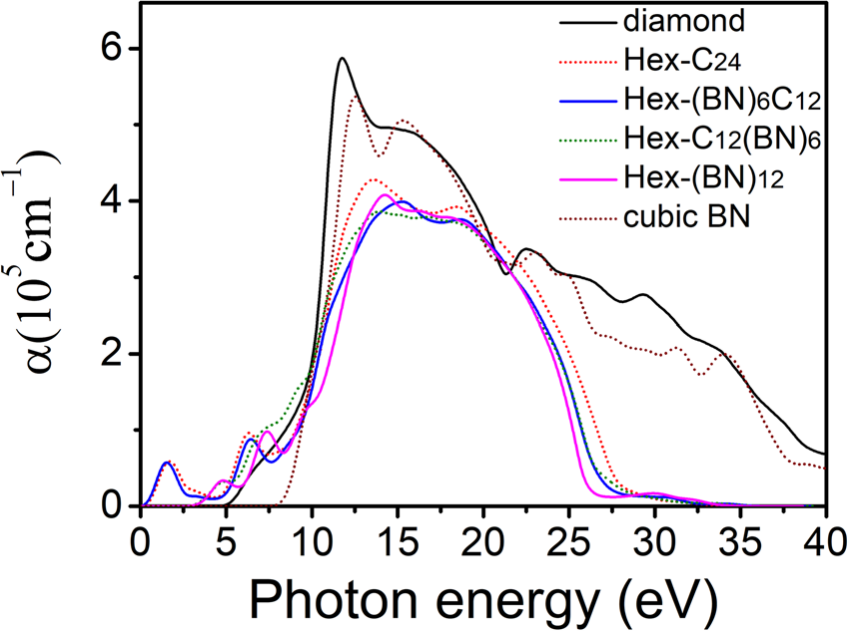
Absorption spectrum of polycrystalline Hex-C_24_, Hex-(BN)_6_C_12_, Hex-C_12_(BN)_6_, and Hex-(BN)_12_. The corresponding values of diamond and cubic BN are shown for comparison.

The absorption coefficient α(ω) of the hexagonal C/BN framework in the polycrystalline structures are shown in Fig. 4. Compared to diamond and cubic BN, the absorption edges of the hexagonal C/BN framework show significant redshifts with a decreasing absorption intensity. For Hex-C_24_ and Hex-(BN)_6_C_12_, the absorption starts at 0 eV while the obvious absorption starts in the infrared region (about 0.60 eV) resulting in small peaks in the low-energy region. The absorption starts in the UV region (3.70 eV) for Hex-C_12_(BN)_6_ and Hex-(BN)_12_. Clearly, the former two have similar spectral curves, and the latter two have similar spectral curves. We attributed this similarity to the sp^2^ hybridization atoms. We deduced that the electronic structure in the region near the Fermi level comes mainly from the sp^2^ hybridized atoms, whereas the contribution from sp^3^ hybridized atoms is very small. The subsequent calculations of electron density of states further confirm this point.

**Figure 5:**
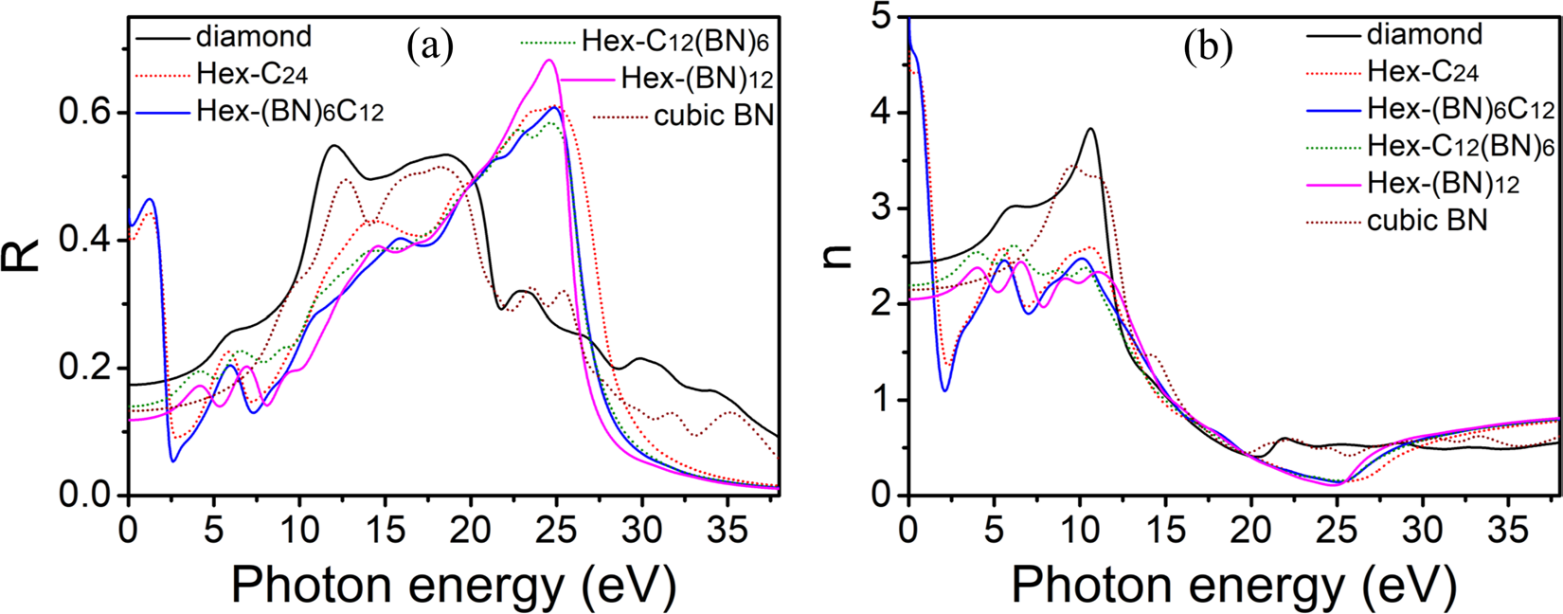
Reflectivity and refractive spectra of polycrystalline Hex-C_24_, Hex-(BN)_6_C_12_, Hex-C_12_(BN)_6_, and Hex-(BN)_12_. The corresponding values of diamond and cubic BN are shown for comparison.

Figure 5 displays the reflectivity and refractive spectra of the poly-crystals for these four hexagonal frameworks. The reflectivity and refractive spectra have peaks in the low and high energy regions, respectively. In the low-energy region, there are comparatively sharp peaks for the reflectivity and the refractive index for Hex-C_24_ and Hex-(BN)_6_C_12_, whereas the reflectivity and the refractive spectrum for Hex-C_12_(BN)_6_ and Hex-(BN)_12_ are relatively flat. In the 10–25 eV energy range, the reflectivity spectra have strong peaks and the refractive spectra show a gradual decrease for all structures. The reflectivity almost reaches zero when the energy exceeds 38.00 eV for all four hexagonal frameworks. The refractive spectrum tends to a stable state when the energy exceeds 30.00 eV. The differences mainly come from the types of atoms with sp^2^ hybridization. We can further infer that if the type of sp^2^ hybridized atoms in the C/BN family is the same, the optical properties will be similar. If they are carbon atoms, the reflectivity and the refractive index will be strong in the low energy range. Therefore, carbon atoms with sp^2^ hybridization are more sensitive to light from the infrared to the visible region than B-N pairs, and the C-C and B-N pairs have a similar sensitivity to high frequencies. The high reflectivity and the low refractive index indicate the possibility of them as anti-ultraviolet radiation materials in this energy range.

**Figure 6:**
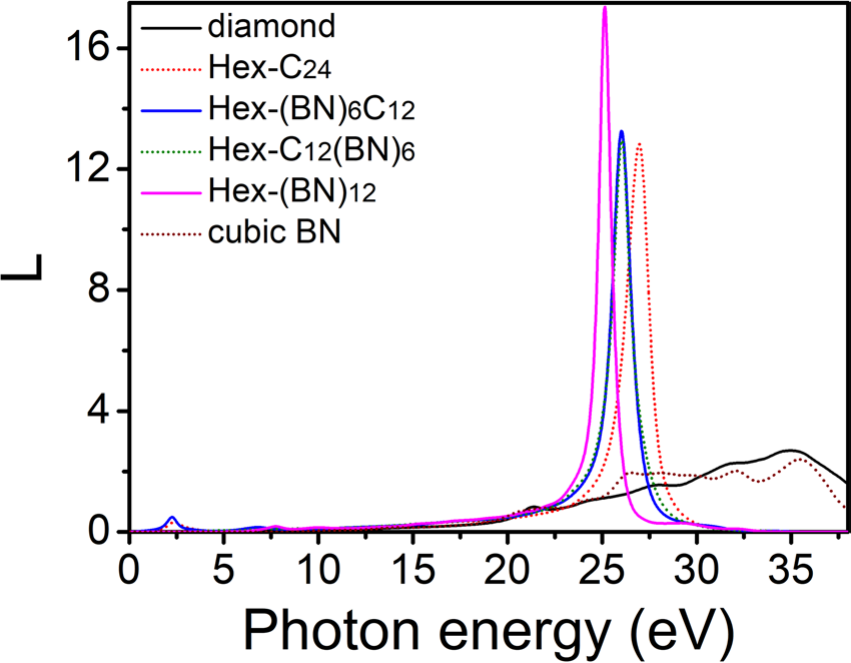
Electron energy-loss spectrum of polycrystalline Hex-C_24_, Hex-(BN)_6_C_12_, Hex-C_12_(BN)_6_, and Hex-(BN)_12_. The corresponding values of diamond and cubic BN are shown for comparison.

Figure 6 shows the energy-loss spectrum and indicates the energy dissipated by the electrons crossing the polycrystalline hexagonal network. The sharp and strong peaks are all concentrated in 23–30 eV energy region. They describe the collective oscillation of the electron cloud relatively to the ionic environment caused by an incident electron passing through the crystal. There is a little redshift in the order of Hex-C_24_, Hex-(BN)_6_C_12,_ Hex-C_12_(BN)_6,_ and Hex-(BN)_12_. The differences in the energy-loss spectrum of these strong peaks are very small. We attributed them to the slight difference in the electronic structure of C-C and B-N bonds. For Hex-C_24_ and Hex-(BN)_6_C_12_, a small peak can be observed in the 1.70–3.00 eV energy range. These sharp and strong peaks can be used to identify the hexagonal family.

**Figure 7:**
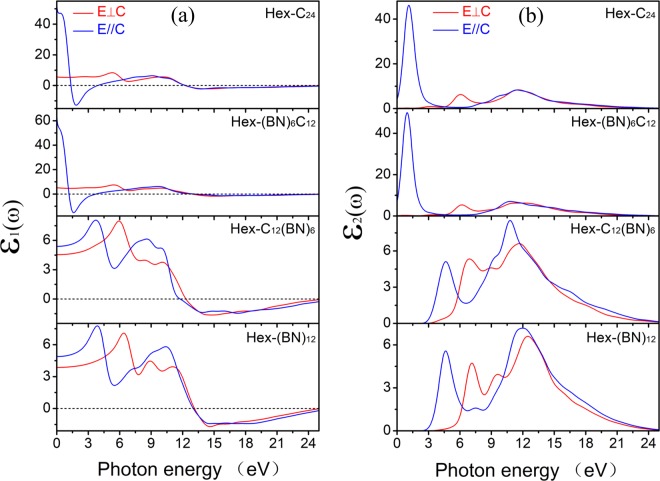
(**a**) Real part and (**b**) imaginary part of the dielectric function of Hex-C_24_, Hex-(BN)_6_C_12_, Hex-C_12_(BN)_6_, and Hex-(BN)_12_ under E⊥C and E//C polarizations.

To study the optical anisotropy of the hexagonal family, the real and imaginary parts of the dielectric functions corresponding to the perpendicular (E⊥C) and parallel (E//C) [0001] directions are considered. Figure 7 shows that the real part and the imaginary part of the dielectric functions of these materials exhibit a general anisotropy. There are relatively strong peaks along the [0001] direction for all four structures. This is related to the anisotropy of the crystal structure and the symmetry along these directions. Even for the same spatial location, the types of atoms are different. Additionally, the same as the dielectric functions of polycrystalline, Hex-C_24_ and Hex-(BN)_6_C_12_, as well as Hex-C_12_(BN)_6_ and Hex-(BN)_12_ have similar spectral curves. By comparing the similarities and the differences for these structures, Hex-C_24_ and Hex-(BN)_6_C_12_ both have carbon atoms with sp^2^ hybridization. The common feature of the other two structures is that the atoms with sp^2^ hybridization are B-N pairs. This also means that the main factors affecting the optical properties of these hexagonal networks are the sp^2^ hybridization atoms.

**Figure 8:**
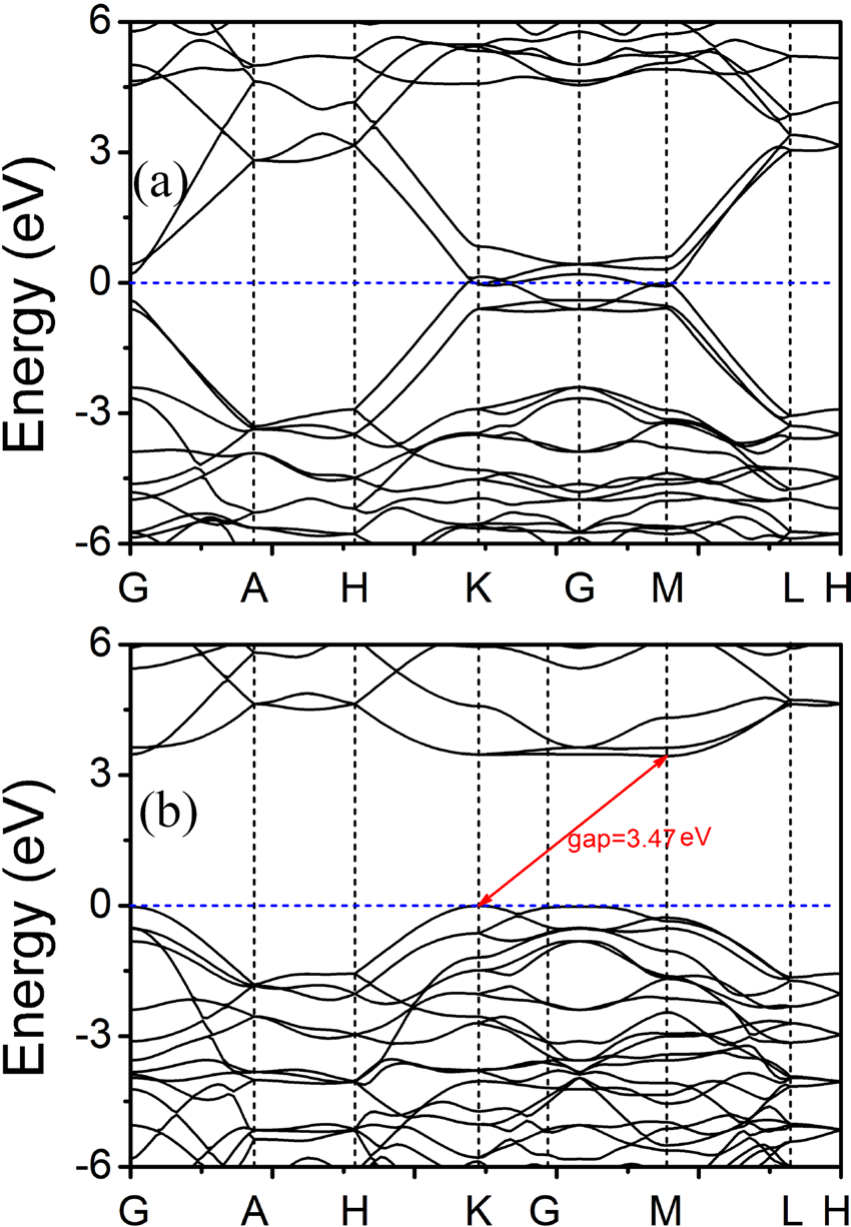
Electronic band structure of (**a**) Hex-(BN)_6_C_12_ and (**b**) Hex-C_12_(BN)_6_. The energy at the Fermi level was set to zero.

**Figure 9:**
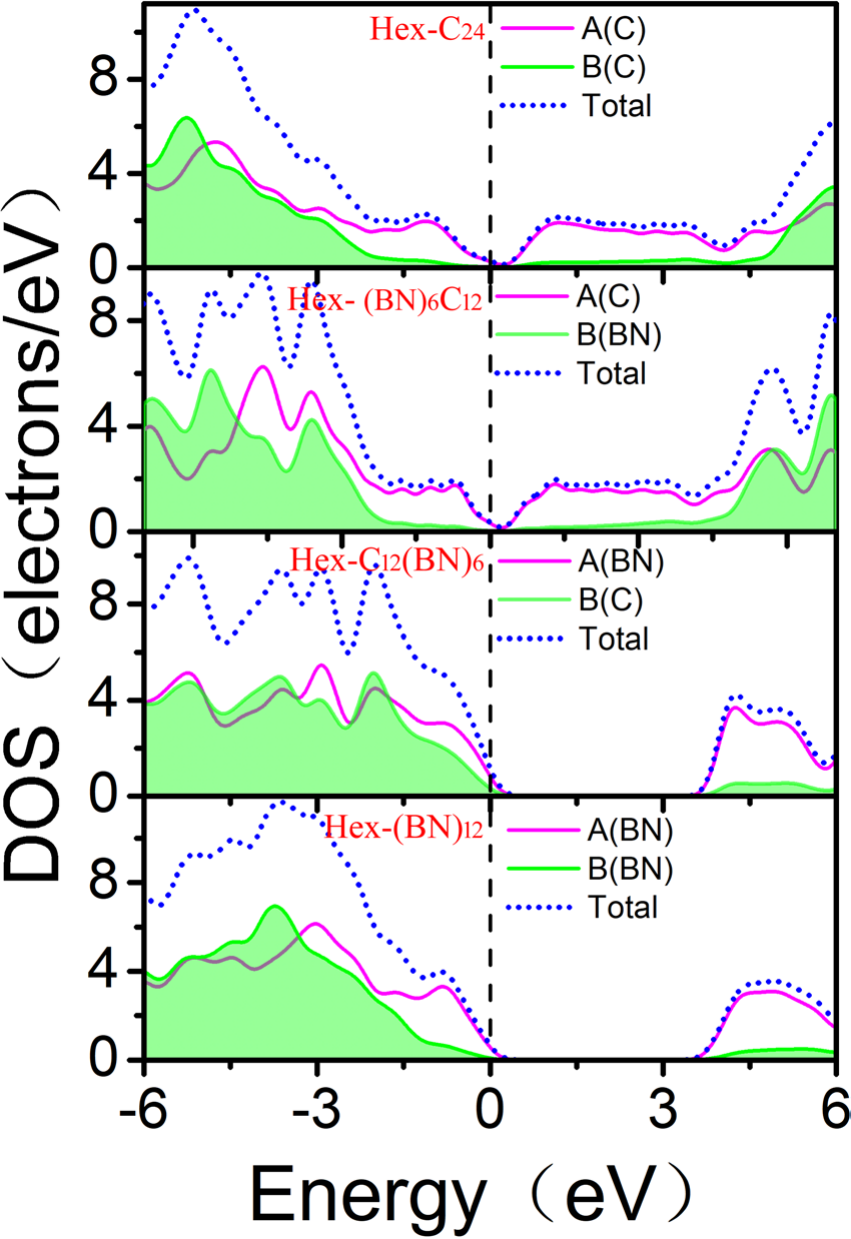
Electron density of states (DOS) projected on the atoms in the A zone with a sp^2^ hybridization and in the B zone with a sp^3^ hybridization, as labeled in Fig. 1(a). The energy at the Fermi level was set to zero.

To further understand the origins of the optical properties, we first determined the band structures of Hex-(BN)_6_C_12_ and Hex-C_12_(BN)_6_, as shown in Fig. 8. Combined with the previous band structures determined for Hex-C_24_ and Hex-(BN)_12_^25,27^, Hex-(BN)_6_C_12_ and Hex-C_24_ have metallic properties, but Hex-C_12_(BN)_6_ and Hex-(BN)_12_ are semiconductors with indirect band gaps of 3.47 and 3.25 eV, respectively. Then, we determined the electron density of states (DOS) projected on the C atoms and the BN atomic pairs at different regions (Fig. 1), as shown in Fig. 9. The metallic properties of Hex-C_24_ and Hex-(BN)_6_C_12_ come from the sp^2^-hybridized C atoms. The electronic states in the region near the Fermi level originate primarily from the sp^2^*-*hybridized atoms in the A zone. The analysis of the spatial distribution of the wave function shows a smaller but significant contribution from the sp^3^*-*hybridized atoms when the atoms in the A zone are B-N pairs. The spatial distribution of the DOS implies that sp^2^-hybridized atoms, either C atoms or B-N pairs, are the main factors affecting the optical and electronic structure in the C/BN materials with sp^2^ and sp^3^ hybridizations according to our results and the existing literature^14,27,30^.

## Discussion

Chemical doping is a common method to control the physical and chemical properties of materials. In systems with C, B and N, all elements have the flexibility of sp, sp^2^ and sp^3^ hybridizations. Different elements and hybrid bonds have different effects on the properties. For instance, the effect of BN-doped carbon materials on the optical properties has become an important aspect in many studies. Here, based on the Hex-C_24_, there are four configurations which correspond to Hex-C_24_, Hex-(BN)_6_C_12_, Hex-C_12_(BN)_6_ and Hex-(BN)_12_. The calculated phonon dispersions confirm the dynamic stability of Hex-(BN)_6_C_12_ and Hex-C_12_(BN)_6_. We systematically studied the optical properties and the roles of the hybrid bonds of a hexagonal C/BN family with sp^2^ and sp^3^ hybridizations using first-principle calculations.

We calculated the dielectric function, the absorption coefficient (α(ω)), the reflectivity(R(ω)), the refractive index ((n(ω)), the energy-loss (L(ω)), the band structures and the DOS projected on atoms with sp^2^ and sp^3^ hybridizations. Our results show that the complex dielectric functions shift from the infrared to the UV region. The absorption starts in the infrared region for Hex-C_24_ and Hex-(BN)_6_C_12_ and in UV region for Hex-C_12_(BN)_6_ and Hex-(BN)_12_. The reflectivity and the refractive index spectra show that carbon atoms with sp^2^ hybridization are more sensitive to light from the infrared to the visible region than B-N pairs, and that the C atoms and the B-N pairs have a similar sensitivity to high frequencies. The sharp and strong peaks in the energy-loss spectrum can be used to identify the hexagonal family. The real part and the imaginary part of the dielectric functions show a significant anisotropy for different polarizations. All calculations show that Hex-C_24_ and Hex-(BN)_6_C_12_ have similar optical properties while Hex-C_12_(BN)_6_ and Hex-(BN)_12_ have similar optical properties. This is because the main factors affecting the optical properties of the hexagonal network are the sp^2^ hybridization atoms, which is also shown by the electronic states near the Fermi level. In addition, our results as well as previously published ones indicate that sp^2^-bonded atoms are the main factors that affect the optical and electronic structures in C/BN materials with sp^2^ and sp^3^ hybridizations. We believe that the results will provide new insight into the optical properties of C/BN materials containing sp^2^- and sp^3^-hybridized C atoms and B-N pairs.

## Methods

We performed first-principle calculations using the CASTEP package based on the density functional theory (DFT)^31,32^. The electron–electron interaction was modeled using a generalized gradient approximation (GGA) from Perdew *et al*.^33^. An energy cut-off of 400 eV was set for the plane-wave basis within a Vanderbilt ultra-soft pseudopotential^34^. A k-point grid for the Brillouin zone in the reciprocal space was set to 0.02 Å^−1^ according to the Monkhorst–Pack method^35^. The lattice constants and the internal coordinates were optimized using the Broyden–Fletcher–Goldfarb–Shanno (BFGS) minimization scheme^36^.

The optical properties can be theoretically calculated based on the complex dielectric function ${{\rm{\varepsilon }}}_{(\omega )}={{\rm{\varepsilon }}}_{1(\omega )}+i{{\rm{\varepsilon }}}_{2(\omega )}\,$^37^, where ω is the frequency of the incident photon. Its imaginary part at longer wavelengths is estimated according to:

$${\varepsilon }_{2}(\omega )=\frac{2\pi {e}^{2}}{{\Omega }_{{\varepsilon }_{0}}}\sum _{{\boldsymbol{k}},c,v}{|{\Psi }_{{\boldsymbol{k}}}^{c}|{\bf{u}}\cdot {\bf{r}}{|\Psi }_{{\boldsymbol{k}}}^{v}|}^{2}\delta ({E}_{{\boldsymbol{k}}}^{c}-{E}_{{\boldsymbol{k}}}^{v}-h\omega )$$

where Ω is the volume of the supercell, and *ε*_0_ is the free-space dielectric constant. $c\,(v)$ labels the conduction (valence) bands, ${\bf{u}}$ labels the polarization direction, ${\bf{r}}$ defines the position vector of the electromagnetic wave, and ${\boldsymbol{k}}$ is the reciprocal lattice vector. The real part of the complex dielectric function can be derived from the imaginary part via the Kramers-Kroning relationships^38,39^. From ${{\rm{\varepsilon }}}_{1(\omega )}$ and ${{\rm{\varepsilon }}}_{2(\omega )}$, the optical properties of the absorption coefficient (α(ω)), the reflectivity (R(ω)), the refractivity index (n(ω)), and the energy-loss (L(ω)) can be determined as^40,41^:

$$\begin{array}{ccc}{\rm{\alpha }}({\rm{\omega }}) & = & \sqrt{2}\omega \sqrt{\sqrt{{\varepsilon }_{1}^{2}(\omega )+{\varepsilon }_{2}^{2}(\omega )}-{\varepsilon }_{1}(\omega )}\\ {\rm{R}}({\rm{\omega }}) & = & {|\frac{\sqrt{{\varepsilon }_{1}(\omega )+i{\varepsilon }_{2}(\omega )}-1}{\sqrt{{\varepsilon }_{1}(\omega )+i{\varepsilon }_{2}(\omega )}+1}|}^{2}\\ n(\omega ) & = & \frac{\sqrt{\sqrt{{\varepsilon }_{1}^{2}(\omega )+{\varepsilon }_{2}^{2}(\omega )}+{\varepsilon }_{1}(\omega )}}{\sqrt{2}}\\ {\rm{L}}({\rm{\omega }}) & = & \frac{{\varepsilon }_{2}(\omega )}{{\varepsilon }_{1}^{2}(\omega )+{\varepsilon }_{2}^{2}(\omega )}\end{array}$$

The optical properties are calculated for both polycrystalline and polarized light.
